# Can proton pump inhibitors increase the risk of sudden unexpected death in Parkinson's disease (SUDPAR)?

**DOI:** 10.1016/j.clinsp.2025.100721

**Published:** 2025-07-31

**Authors:** Francisco Sandro Menezes-Rodrigues, Fernando Sabia Tallo, Afonso Caricati-Neto, Josef Finsterer, Carla Alessandra Scorza, Fulvio Alexandre Scorza

**Affiliations:** a Universidade Federal de São Paulo (UNIFESP), São Paulo, SP, Brazil; b Klinikum Landstrasse, Messerli Institute, Vienna, Austria

Parkinson’s Disease (PD), a leading neurodegenerative disorder strongly associated with aging, has shown a worrisome rise in mortality rates over the past decade, emerging as an increasingly urgent public health concern.^1^^,^^2^ Current studies indicate that individuals with PD exhibit a higher incidence of premature death when compared to healthy controls, commonly resulting from complications such as pneumonia, cerebrovascular events, and cardiovascular disorders. More recently, a significant number of deaths in PD patients have been attributed to a phenomenon termed Sudden Unexpected Death in Parkinson’s Disease (SUDPAR).^1^, ^2^, ^3^ Nevertheless, general medical care for individuals with Parkinson’s Disease (PD) and Parkinsonism (PS) rarely incorporates the growing body of evidence implicating primary cardiac dysfunction.^1^, ^2^, ^3^

Gastrointestinal complications, including constipation, dysphagia, and Gastroesophageal Reflux Disease (GERD), are frequently reported in the clinical trajectory of most PD patients.^4^ GERD, a globally prevalent gastrointestinal disorder affecting populations across both developed and developing nations, results from the retrograde flow of gastric acid into the esophagus.^5^, ^6^, ^7^ Among the various pharmacological options for managing GERD, Proton Pump Inhibitors (PPIs) ‒ also known as antisecretory agents ‒ remain the most widely prescribed due to their ability to suppress acid secretion by parietal cells.^8^ In light of these data, it becomes imperative to examine the potential exacerbation of SUDPAR in PD patients with concurrent GERD who are treated with PPIs such as omeprazole and pantoprazole. These agents have been implicated in increasing cardiovascular risk through mechanisms involving reduced systemic vasodilation, elevated blood pressure, and negative inotropic effects. Such adverse outcomes may further elevate the likelihood of neurological, renal, and cardiovascular complications in this vulnerable population.^9^, ^10^

PPIs have been shown to attenuate Nitric Oxide (NO) synthesis by inhibiting Endothelial Nitric Oxide Synthase (eNOS) and reducing Bradykinin (BK)-induced NO production. Additionally, omeprazole impairs the formation of prostaglandin I^2^ metabolites, particularly 6-keto-prostaglandin 1α. The reduction in BK-induced eNOS phosphorylation observed with omeprazole administration suggests a decrease in NO bioavailability, likely mediated by previously proposed mechanisms such as suppressed eNOS synthesis or accumulation of intracellular Asymmetric Dimethylarginine (ADMA).^9^^,^^11^^,^^12^ This is attributed to PPIs’ inhibition of Dimethylarginine Dimethylaminohydrolase 1 (DDAH1), the enzyme responsible for degrading ADMA. Elevated ADMA concentrations in plasma are associated with increased mortality and heightened cardiovascular risk.^13^^,^^14^ However, findings from a pilot, open-label, crossover study by Ghebremariam et al..^14^ Involving coronary artery disease patients and healthy subjects revealed no significant alterations in vascular endothelial function following PPI administration. Similarly, Tomasi et al. reported no changes in plasma ADMA levels at clinically relevant PPI dosages, raising questions about the clinical relevance of DDAH1 inhibition as a plausible mechanism for PPI-associated cardiovascular risk.^15^, ^16^

Beyond NO pathway inhibition, recent evidence indicates that *H*^+^/*K*^+^-ATPase is expressed both transcriptionally and at the protein level in cardiac tissue of rats.^17^ Physiological and biochemical data further support the presence of a cardiac-specific *H*^+^/*K*^+^-ATPase,^17^, ^18^ Beisvag et al.,^18^ postulated that this enzyme could be involved in maintaining myocardial potassium and hydrogen ion homeostasis. Therefore, inhibition of cardiac *H*^+^/*K*^+^-ATPase might induce cellular acidosis, a known factor in the attenuation of cardiac contractility, particularly through decreased myofilament responsiveness to intracellular calcium [Ca^2+^],^19^ Nevertheless, a recent investigation showed that administration of a high-dose pantoprazole regimen (80 mg bolus over two minutes followed by 8 mg/h intravenously) did not significantly impair hemodynamic parameters or left ventricular function in healthy volunteers.^20^ This regimen is frequently employed to prevent rebleeding following endoscopic management of peptic ulcer hemorrhage.^21^

Given the findings of Potnuri et al.,^22^ who demonstrated that histamine acts via H_2_ Receptors (H_2_R) in the myocardium of spontaneously hypertensive rats ‒ thereby contributing to adverse cardiac remodeling ‒ it is reasonable to suggest that famotidine may represent a safer antisecretory alternative in patients at elevated cardiovascular risk, including those with PD at risk for acute myocardial infarction and hypertension. The same authors showed that H_2_R blockade by famotidine improved cardiac function and attenuated left ventricular hypertrophy, effects comparable to those induced by metoprolol. These results were corroborated by experiments showing hypertrophy in cultured cardiomyocytes exposed to H_2_R agonists such as histamine and amthamine. Taken together, these findings support the notion that H_2_R antagonists may offer a cardioprotective and safer therapeutic option for PD patients at risk of SUDPAR.

Another therapeutic class for GERD management, recently introduced and potentially safer from a cardiovascular standpoint in PD patients, comprises the Potassium-Competitive Acid Blockers (PCABs). Unlike PPIs, these agents do not interfere with NO production, NO-mediated vasodilation, or myocardial contractility. Among them, vonoprazan appears to be a promising candidate for GERD treatment in PD patients, despite the current lack of targeted studies validating its safety in this population. A recent study revealed that PCAB administration effectively prevented upper gastrointestinal bleeding in patients undergoing Percutaneous Coronary Intervention (PCI) and receiving antithrombotic therapy, without compromising coronary blood flow.^23^ These findings suggest that PCABs may serve as a suitable option for PD patients requiring acid suppression therapy.

The study by de Araújo et al.,^24^ provided compelling evidence that intravenous administration of omeprazole (10 mg/kg) prior to Cardiac Ischemia and Reperfusion (CIR) in Wistar rats led to a higher incidence of ventricular arrhythmias, atrioventricular block, and mortality, similar to outcomes observed with Methylene Blue (MB), another agent known to inhibit the NO pathway. Moreover, the combination of OME and MB prior to CIR resulted in increased myocardial injury and elevated serum creatine kinase-MB levels, reinforcing the hypothesis that OME may induce or aggravate cardiovascular damage, particularly in patients at risk of SUDPAR.

Given the accumulating evidence linking PPI use to mechanisms that may adversely affect cardiovascular and autonomic regulation, especially in patients with PD, the potential for these drugs to increase the risk of Sudden Unexpected Death in Parkinson's Disease (SUDPAR) cannot be overlooked. While current data remain partially contradictory, and further investigation is essential, clinicians should exercise caution when prescribing PPIs to PD patients, particularly those with preexisting cardiovascular risk. Safer alternatives, such as H_2_ receptor antagonists or PCABs, may represent more appropriate options in this high-risk population.

## Conflicts of interest

The authors declare no conflicts of interest.
